# Why does uptake of family planning services remain sub-optimal among Nigerian women? A systematic review of challenges and implications for policy

**DOI:** 10.1186/s40834-020-00133-6

**Published:** 2020-10-31

**Authors:** Ifeyinwa Chizoba Akamike, Ijeoma Nkem Okedo-Alex, Irene Ifeyinwa Eze, Obumneme Benaiah Ezeanosike, Chigozie Jesse Uneke

**Affiliations:** 1 Department of Community Medicine, Alex Ekwueme Federal University Teaching Hospital, Abakaliki, Nigeria; 2 African Institute for Health Policy and Health Systems, Ebonyi State University, Abakaliki, Nigeria; 3 Department of Paediatrics, Alex Ekwueme Federal University Teaching Hospital, Abakaliki, Nigeria

**Keywords:** Family planning, Contraception, Challenges, Barriers, Policy implications

## Abstract

**Background:**

Over the years, family planning uptake in Nigeria has remained low and this is as a result of the various challenges and barriers faced by women. The aim of this study was to systematically review studies on family planning services undertaken in Nigeria in order to understand the challenges to uptake of the services and the policy implications.

**Methods:**

A PubMed search was performed in June 2020 and studies that investigated challenges of family planning uptake in Nigeria published in English between 2006 and 2020 were sought. A combination of the search terms family planning, contraceptives, challenges, barriers, Nigeria was used. Review articles, case reports, and case studies were excluded. Studies that did not report barriers or challenges to family planning or contraceptives were excluded.

**Result:**

Twenty seven studies carried out in Nigeria which provided sufficient information were identified and used for this review. The Uptake of family planning recorded in the reviewed studies ranges from 10.3 to 66.8%. Challenges that are client related include education, desire for more children, uncertainty about its need, partner disapproval, previous side effects, religious beliefs, culture disapproval, age, marital status, and wealth index, residence, ignorance, embarrassment, domestic violence and sexual factor. Health service related factors identified include cost, difficulty accessing services, and procurement difficulties. Recommendations for family planning propram and policy include targeting of health service delivery for improvement, focus on gender issues and male involvement, involvement of religious leaders, targeting of younger women for better education and counseling, and continuous awareness creation and counseling among others.

**Conclusion:**

The review has shown that uptake of family planning remains low in Nigeria and challenges abound. We recommend that strategies that are multi-sectoral should be applied to address the multi-pronged challenges facing uptake of family planning services.

**Supplementary Information:**

**Supplementary information** accompanies this paper at 10.1186/s40834-020-00133-6.

## Introduction

Maternal health outcomes in Nigeria have continued to be of great concern. According to the 2018 NDHS, there were 512 maternal deaths per 100,000 live births, which is still high when compared to the developed countries [1]. Family planning is one of the ways through which maternal deaths can be reduced. The interval between pregnancies can be prolonged by providing family planning services for postpartum women and this can help protect their health and that of their newborns [2]. Efforts have been ongoing to ensure that contraceptives are available to women in Nigeria [3, 4] however, uptake is still low with only 12% of women using a modern method of family planning [1]. There is also high unmet need for family planning in Nigeria with about 19% of married women having an unmet need for family planning [1]. This can subsequently lead to high fertility rates and increased population growth in the face of economic instability facing developing countries [5]. Additionally, maternal mortality and morbidity can be unfavourable to economic development.

In Nigeria, there is a high level of knowledge about family planning but most women still do not make use of family planning services [1]. There are still a number of factors that women point at as reasons for not using a method. Challenges to the uptake of family planning services as identified by previous studies include factors such as spousal disapproval, religious beliefs, cultural disapproval, fertility desires and fear of side effects, long distances of sources, poor services of family planning clinics, limited knowledge and skills of providers, workload at the clinic, inconvenience at the family planning clinic, and cost among others [6–9].

It is necessary to identify and compare these studies based on location and the most frequent factors so as to aid in designing interventions that will be most effective for particular settings.

The purpose of this study was to systematically review studies on family planning services undertaken in Nigeria in order to understand the challenges to uptake of the services and the policy implications.

## Methods

The Preferred Reporting Items for Systematic Reviews and Meta-Analysis checklist for reporting a systematic review or meta-analysis protocol was used for this review [10].(See Additional file 1).

### Search strategy

A systematic review of published quantitative and qualitative literature was carried out. A PubMed search was performed in June 2020. Additional search was also carried out in AJOL. Studies that investigated challenges to uptake of family planning services in Nigeria from 2006 to 2020 were sought. Search terms used include Uptake, family planning, family planning services, contraceptives, challenges, barriers, Nigeria.(See Additional file 2).

### Inclusion criteria

Studies were eligible for inclusion if they were carried out in Nigeria and published in English language, published between the years January 2006 and June 2020, measured barriers/challenges to any form of family planning/contraceptive method; and used any quantitative/qualitative study design.

### Exclusion criteria

Review articles, case reports, and case studies were excluded. Also studies that did not report barriers or challenges to family planning or contraceptives were excluded.

**Fig. 1:**
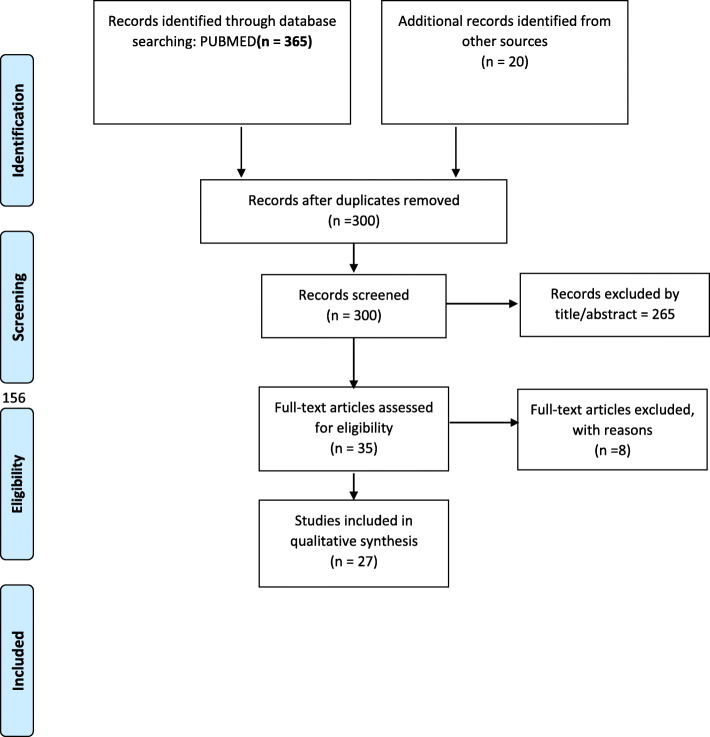
Article selection and inclusion process

The search yielded a total of 385 publications. Citations identified through the search strategy were initially reviewed for inclusion based on information contained in titles, abstracts, citation information, and keywords. Full text articles were obtained for all eligible studies and for those requiring further review to determine eligibility. Those articles that fulfilled the inclusion criteria were critically appraised and included in the review. Figure 1 shows the article selection and inclusion process.

### Data extraction

A data extraction form was developed and used for data extraction. Mendeley reference manager was used to keep track of references. The data extraction form included the following domains: the name of first author and year of publication, study location and setting, study design, contraceptive prevalence rate/uptake, challenges/barriers, and policy implications.

### Quality appraisal

Quality appraisal of the studies included in this review was carried out by two review authors. The Quality Assessment Tool for Observational Cohort and Cross-Sectional Studies was used to assess the quality of the quantitative studies. This quality assessment tool has been used in other systematic reviews [11, 12] (See additional file 3) The tool is made up of 14 questions assessing different aspects of a study including but not limited to definition of objectives, study population, sampling strategy, sample size and statistical analyses. Each question in the tool is scored as Yes (1) or No (0), and others (CD, cannot determine; NA, not applicable and NR, not reported). All the studies included in this review were assessed for quality using the appropriate criteria.

All the studies fulfilled the quality criteria except that four studies did not describe how sample size was calculated [13–16] and eleven studies did not measure and adjust for the impact of key confounding variables [13–15, 17–24].

The CASP qualitative checklist was used to assess the quality of the qualitative studies [25]. The tool has been used in previous studies [26]. The tool has 10 questions assessing different aspects of a qualitative study including research design, recruitment strategy, data collection method, ethical issues, data analysis, and reporting of findings. Each question has three options which are “Yes”, “No”, and “Can’t tell”. All the qualitative studies included in this review fulfilled the CASP criteria. (See additional file 4).

## Result

### Search results

Twenty Seven studies carried out in different states in Nigeria which provided sufficient information were identified and used for this review. Three of the studies were carried out in northern part of the country [21, 27, 28], four were carried out in the Southern part [16, 19, 29, 30], four were carried out in Eastern part [13, 15, 31, 32], seven were carried out in the western part[17, 18, 23, 24, 33–35]while nine studies were carried out in more than one region of the country [14, 20, 22, 36–41]. All the studies reviewed were cross sectional studies, among which were two secondary data analyses [36, 37] and four qualitative studies [27, 28, 40, 41](Table 1).

**Table 1:** Summary of all studies

Author/Year of publication	Type of study	Location of study	Uptake/contraceptive prevalence	Factors/challenges to uptake
Oye-Adeniran/2006 [22]	Cross-sectional study	Anambra, Oyo, Kaduna & Bauchi	22.1%	Client and Health service related
Okanlawon/2010 [23]	Cross-sectional	Ogun	31.6%	Client and Health service related
Nwachukwu/2008 [15]	Cross sectional	Imo	29.7%	Client related and Health service related
Umoh/2011 [29]	Cross-sectional	Akwa-Ibom	52.6%	Client related
Idris/2013 [21]	Crosss-sectional	Kaduna	14%	Client related
Asekun-Olarinmoye/2013 [24]	Cross-sectional study	Osun	13.1%	Client related
Eluwa/2016 [20]	Cross-sectional	Anambra, Abia, Delta, Ebonyi, Edo, Enugu	41%	Client related
Ugboaja/2011 [31]	Cross-sectional	Anambra	44%	Client related
Egede/2015 [13]	Cross-sectional	Ebonyi	28.3%	Client related
Adebayo/2016 [35]	Cross-sectional	Ondo	66.8%	Client related
Durowade/2017 [17]	Cross-sectional	Ekiti	50.5%	Client related
Solanke/2017 [37]	Cross-sectional study (secondary data analysis)	Nigeria	19.8%	Client related
Etokidem/2017 [30]	Cross-sectional	Cross-River	17.2%	Client related
Okigbo/2014 [16]	Cross sectional	Cross River	25.9% (among ART clients), 15.8%(among HCT clients)	Client Related
Adebowale 2011 [36]	Cross sectional (Secondary data analysis)	Nigeria	26%	Client Related
Schwandt/2017 [14]	Cross-sectional	Abuja, Benin, Ibadan, Ilorin, Kaduna, Zaria	NA	Health service related
Akamike/ 2019 [32]	Quasi-experimental	Ebonyi	9.9% in intervention arm, 12.8% in control arm	Client and Health Service related
Abiodun /2015 [33]	Cross sectional	Ogun	54.1% (EC)	Client related
Adefalu/2019 [27]	Qualitative	Kebbi and Sokoto	NA	Client and Health service related
Adeyemi/2016 [34]	Cross sectional	Oyo	25.4%	Client related
Bishwajit/2018 [38]	Secondary data Analysis	Nigeria	16%	Client Related
Chimah/ 2016 [18]	Cross sectional	Lagos	31.1%	Client related
Johnson 2/017[39]	Secondary data analysis	Nigeria	10.3%	Client related
Omo-Aghoja/2009 [19]	Cross sectional	Delta	29%	Client related
Sinai /2019 [28]	Qualitative	Kaduna	NA	Client and health service related
Ankomah /2013 [40]	Qualitative	13 States in Nigeria	NA	Client and Health worker related
Aransiola /2014 [41]	Qualitative	Ibadan and Kaduna	NA	Client related

*EC* Emergency contraception, *ART* Antiretroviral, *HCT* HIV counseling & Testing.

### Study characteristics

For the purpose of this review, the studies were divided into three categories. The first category consisted of studies that identified only client related challenges to family planning. The second category are studies that identified health service related challenges while the third category identified both client and health service related challenges to uptake of family planning.(Table 1).

Nineteen studies [13, 16–21, 24, 29–31, 33–39, 41] identified only client related factors, seven studies identified client and health service related factors [15, 22, 23, 27, 28, 32, 40] while one study identified only health service related factor [14].

#### Uptake of family planning services

Uptake of family planning ranged from 10.3% to 66.8%. Only three studies reported uptake of greater than 50% [17, 29, 35]. (Table 1).

### Health service related title

**Health service related factors** identified include cost of services in four studies [15, 28, 32, 40], and difficulty accessing services in two studies [14, 23]. Procurement difficulties, long distances of sources, and poor service of family planning clinics were all reported in one study. Prior counseling was reported in one study [22]. Prior counseling was shown to significantly improve the continuation rate of contraception. Health facility dependence on donor organizations for supply of FP materials and women being responsible for purchasing required consumables were also identified as factors that affect uptake of family planning [27]. Stock out and providers adherence to cultural practices were reported in one of the studies [28].(Table 2).

**Table 2:** Challenges of uptake of family planning services

Author/Year of publication	Location of study	Factors/challenges to uptake
Oye-Adeniran/2006	Anambra, Oyo, Kaduna & Bauchi	**Client related factors**:fear of side effects, religious belief, older age, marital status and education.
		**Health service related factor:** Prior counseling significantly improved the continuation rate of contraception)
Okanlawon/2010	Ogun	**Health service related**: difficulty in accessing family planning services. **Client related:** misperceptions that contraceptives are dangerous)
Umoh/2011	Akwa-Ibom	**Client related factors**: education, side effect, uncertainty about its need, partner objection
Idris/2013	Kaduna	**Client related factors**: desire for more children, religion, partner disapproval, lack of privacy
Asekun-Olarinmoye/2013	Osun	**Client related factors:** Desire for more children Fear of side effects/complications, ignorance, perceived low-risk of getting pregnant/not sexually active, Religion, Partner disapproval, marital status, number of living children, loss of a child and awareness of place of family planning services
Eluwa/2016	Anambra, Abia, Delta, Ebonyi, Edo, Enugu	**Client related:** lower education, higher number of children, being single
Ugboaja/2011	Anambra	**Client related:**(fear of side effects, not aware of where to access services, husband’s disapproval, cultural unacceptability, religious disapproval
Egede/2015	Ebonyi	**Client related factors**: desire for more children, religious prohibition, spousal disapproval, perceived side effects
Adebayo/2016	Ondo	**Client related factors:** education, health condition
Durowade/2017	Ekiti	**Client related factors:** desire for more children, partner disapproval, fear of side-effects, culture, ignorance, marital status, educational level and religion.
Schwandt/2017	Abuja, Benin, Ibadan, Ilorin, Kaduna, Zaria	**Health service related factors:** Restriction of access due to age, parity, marital status
Solanke	Nigeria	**Client factors**: lower education, higher number of living children, being single
Etokidem/2017	Cross-River	**Client related factors:** religious beliefs, cultural barrier desire for more children, partner disapproval, family planning does not work, it reduces sexual enjoyment, it promotes unfaithfulness/infidelity
Okigbo, 2014	Cross River	**Client Related:** Partner opposition
Adebowale 2011	Nigeria	**Client Related:** Education, wealth index, region, religion, completed fertility,
Nwachukwu 2008	Imo	**Client related:** fear of the unknown effects, spouse’s disapproval, religious belief, cultural disapproval, shyness, moral disapproval, ignorance. **Health service related**: cost, procurement difficulties, long distances of sources, and poor service of Family Planning Clinics
Akamike 2019	Ebonyi	**Client related:** Desire for more children, fear of side effects, partner opposition, religious opposition, ignorance, cultural unacceptability, unaware of where to access services **Health service related:** cost
Abiodun 2015	Ogun	**Client related:** Age Region/Residence
Adeyemi 2016	Oyo	**Client Related:** younger age group, less educated, singles,
Bishwajit 2018	Nigeria	**Client related:** Domestic violence
Chimah 2016	Lagos	**Client Related:** Lack of fund, Too embarrassed to source for it, fear of side effects, fear of adult disapproval, Ignorance, Unexpected timing of sexual intercourse
Johnson 2017	Nigeria	**Client related:** Education, wealth quintile, age, residence
Omo-Aghoja 2009	Delta	**Client related:** Fear of side effects, lack of knowledge, spousal disapproval, culture, Religion, Desire for more children
Adefalu 2019	Kebbi and Sokoto	**Client related:** Influence of relatives, Religion, Ignorance, Number of male children, Age at marriage, Educational attainment, spousal rejection of family planning. **Health service related**: health facility dependence on donor organizations for supply of FP materials, women seeking to accept contraceptive are responsible for purchasing required consumables
Sinai 2019	Kaduna	**Client related**: Religion, Desire for more children, poor community support, partner disapproval **Health service related:** Cost, Stockout, providers adhering to cultural practices
Ankomah 2013	13 States in Nigeria	**Client related** Fear of side effects, causes promiscuity, religion, lack of social support, lack of family support, lack of husband’s support **Health service related:** Cost
Aransiola 2014	Ibadan and Kaduna	**Client related**: Lack of partner support

### Client related factors

Challenges that are client related include education, desire for more children, uncertainty about the need for family planning, partner disapproval, previous side effects, religious beliefs, culture disapproval, age, marital status, wealth index, residence, ignorance, shyness, domestic violence and sexual factor. (Table 2).

#### Misconceptions about side effects

Twelve studies reported fear of side effects or misperception that family planning is dangerous as reasons for non-use of family planning services [13, 15, 17–19, 22–24, 29, 31, 32, 40].

#### Education

Nine studies revealed lower education as a barrier to use of family planning services [17, 20, 22, 29, 34–37, 39].

#### Number of children

The desire to have more children was shown to affect use of family planning services. Some studies reported having not completed their family size as a factor [13, 17, 19, 21, 24, 28, 30, 32] while some reported that people with more children were more likely to use a family planning method [20, 24, 37]. Number of male children was also reported in one study to affect uptake of family planning. Having desired number of male children encouraged uptake of family planning [27].

#### Uncertainty about its need

Two studies reported uncertainty about the benefits of family planning as a reason for not using a method [29, 30]**.**

#### Partner disapproval

Fifteen studies reported spousal/partner disapproval as a barrier to the use of family planning services [13, 15–17, 19, 21, 24, 27–32, 40, 41].

#### Religious beliefs

Religious belief was reported in 14 studies as a barrier to uptake of family planning [13, 15, 17, 19, 21, 22, 24, 27, 28, 30–32, 36, 40].

#### Cultural disapproval

Cultural and moral disapproval were reported in six studies [15, 17, 19, 30–32].

#### Influence of relatives/lack of social support

Influence of relatives was documented as a barrier to uptake of family planning in two studies [27, 40], while poor support of community or lack of social support was reported as a factor that affects uptake of family planning in two studies [28, 40].

#### Ignorance of family planning/where to access services

Ignorance about family planning was reported in six studies [15, 17, 18, 24, 27, 32]. Three studies reported lack of awareness of where to access family services as a barrier to use of family planning methods [24, 31, 32].

#### Sexual factor

Reduction in sexual enjoyment [30] and promotion of unfaithfulness/infidelity [30, 40] were also documented as barriers to use of family planning methods. Another study reported unexpected timing of sexual intercourse as a barrier to use of contraceptives [18].

#### Age

Some of the studies reported age as a factor that affects uptake of family planning services. Three studies found that older women were more likely to use a method of family planning [22, 34, 39], while another study among university students reported that younger women were more likely to use emergency contraceptives [33]. Age at marriage was documented by one study as a factor that affects uptake of family planning. Women who married at an early age were said to be more likely to use contraceptives [27].

#### Marital status

Four studies reported that people who were married were likely to use a method of family planning [17, 22, 24, 34]. Two studies reported being single as a barrier to family planning uptake [20, 37]. One study also showed perceived low-risk of getting pregnant or not being sexually active as a barrier to uptake of family planning methods [24].

#### Wealth index

Wealth index was reported in two studies [36, 39]. Poorer women were reported to be less likely to use a method of family planning.

#### Region/residence

Region was reported as a factor that affects family planning uptake. In two studies, rural residence was a barrier to uptake [36, 39], while in another study, rural residence was a predictor of uptake [33].

#### Domestic violence

One study reported an association between domestic violence and use of contraceptives [38].

#### Embarrassment

Two studies reported that respondents did not use a method of contraception because they felt embarrassed to go for it [15, 18].

### Implications for family planning program and policy

A number of recommendations for improving uptake of family planning services were highlighted in the included studies.. (Table 3) These include:

1. Awareness creation through training [22], education [15, 23], institution of community based behavioural change communication program [24], and targeted campaign for counseling [13, 29, 31]
2. Programs targeting service delivery such as improving quality of maternal health services [21], and scaling up services [20]. One study recommended that more primary health centers, with strong family planning facilities should be made available in the rural areas [15]. It was also recommended that Policies that allow larger pool of providers to be available in all channels, such as task shifting should be positioned to address the problem of inadequate manpower plaguing the public health system in the Northern part of the country and the power of social networks in influencing reproductive health should also be explored [27].
3. Engagement of religious leaders. One Study recommended that religious leaders should be targeted for more education on the benefits of Modern Birth Control Method use [15].
4. Programmes targeting gender issues, male involvement and culture: One of the studies recommended that programs/policies that consider gender and cultural influence on family planning service utilization and method uptake should be modified. There is also a need to increase the self-efficacy of the clients towards contraceptive use and spousal communication about family planning in general [16, 30]. Some of the studies recommended that the influence of the male partner should also be considered, and more male friendly services should be incorporated into the practice of family planning [17, 32, 34, 41].It was also recommended in one study that policy makers place special emphasis on developing strategies to protect women from any form of perpetration of domestic violence and to integrate gender issues to matters that concern women’s reproductive health [38]
5. Increased political and financial buy-in, especially at the state and local government levels was also recommended in one study [27]. One of the studies recommended that family planning interventions should concentrate on the benefits of family planning at the family level and not at the state or national level [40].
6. Programmes targeted at younger women/youths: Two studies recommended that efforts should be intensified to improve knowledge about contraceptives and to promote safe sexual practice including effective contraceptive use among young people in secondary schools and tertiary institutions [18, 33].

**Table 3:** Policy implications

Author/Year	Policy implications
**Oye-Adeniran/2006**	Health-care providers should be trained to offer counseling services to all clients in general, and young, unmarried and uneducated women in particular in order to improve their acceptance of contraceptives.
**Okanlawon/2010**	Findings may aid in development of targeted interventions to educate refugee youths in order to dispel misconceptions about the safety of contraceptives and ensure adequate access to family planning services
**Umoh/2011**	There’s need to tackle known obstacles to contraceptive uptake. Also targeted campaign and every available opportunity should be used to provide reproductive counselling to women especially on contraception
**Idris/2013**	It is recommended that while there is need to raise awareness on the utilisation of maternal health services, bring it closer to the mothers and make it more affordable, there is a more pressing need to improve its quality, especially through the alleviation of negative attitude of health care providers.
**Asekun-Olarinmoye/2013**	It is recommended that a community-based behavioral-change communication program be instituted, aimed at improving the awareness and perceptions of women with respect to desire for more children, at bridging the knowledge gaps about contraceptive methods, and at changing the deep-seated negative beliefs related to contraceptive use in Nigeria
**Eluwa/2016**	Scale-up of postpartum IUD services is a promising approach to increasing uptake of long-acting reversible contraceptives among women in Nigeria.
**Ugboaja/2011**	The use of more reliable methods should be encouraged through sensitization campaigns
**Egede/2015**	More education and campaigning is still needed to improve the presently low prevalence and utilization of contraception
**Adebayo/2016**	Concerted effort at increasing uptake is advocated to bridge the gap between client counselling and uptake.
**Durowade/2017**	The campaigns for family planning services should aim at the misconceptions in order to drive the demand and remove the barriers. The influence of the male partner should also be considered, and more male friendly services should be incorporated into the practice of family planning
**Schwandt/2017**	A constellation of creative interventions aimed at reducing, and eventually eliminating provider imposed restrictions to family planning use in Urban Nigeria are needed urgently to make family planning truly accessible
**Solanke**	The scope, content, and coverage of existing BCC messages should be extended to cover the contraceptive needs and challenges of women of advanced reproductive age in the country.
**Etokidem/2017**	The findings of this study suggest that family planning uptake would increase if couples make joint decision in this regard. There is also a need to ensure a change of behavior and attitude.
**Okigbo, 2014**	There is need to modify existing programs or to develop new programs/policies that consider gender and cultural influence on family planning service utilization and method uptake. There is a need to increase the self-efficacy of the clients towards contraceptive use and spousal communication about family planning in general
**Adebowale 2011**	Understanding the mechanisms that underline the relationships between contraceptive use and demographic characteristics are crucial in designing effective public policies aimed at improving maternal health.
**Nwachukwu 2008**	Governments and NGOs should provide more educational opportunities in the rural areas for the purpose of teaching birth control methods. More primary health centers, with strong family planning facilities should be made available in the rural areas. It is also necessary for religious leaders to be targeted for more education on the benefits of Modern Birth Control Method use.
**Akamike 2019**	Community-based interventions such as training of community resource persons particularly men is of great importance in improving uptake of health services
**Abiodun 2015**	There is a need for targeted health education campaign to promote consistent and proper condom use among young people. It is important that programmes and policies should be engen- dered to improve the knowledge of university students and address misconception about EC. There is also a need to deliberately engage health workers in the promotion of EC and in making the commodities readily accessible to university students and young people in general
**Adeyemi 2016**	There needs to be a conscious effort to educate women about contraception and encourage its use. Reproductive health programs and policies should adequately involve male partners/men. Researchers should seek to explore all avenues to make contraception a “couple thing” from inception
**Bishwajit 2018**	It is recommended that policy makers place special emphasis on developing strategies to protect women from any form of perpetration and to integrate gender issues to matters that concern women’s reproductive health
**Chimah 2016**	Efforts should be intensified to promote safe sexual practice including effective contraceptive use among secondary school students.
**Johnson 2017**	Measures should be taken to improve female literacy and employment
**Omo-Aghoja 2009**	Effective educational and counseling interventions are likely to improve knowledge and uptake
**Adefalu 2019**	Increased political and financial buy-in, especially at the state and local government levels, needs to be developed. Policies that allow larger pool of providers to be available in all channels, such as task shifting should be positioned to address the problem of inadequate manpower plaguing the public health system in the North.The power of social networks in influencing reproductive health, may serve as an effective route of effecting FP behavioral change.
**Sinai 2019**	Programmatic interventions at home, in the community and at the facility should capitalise on the changing cognitive and emotional ideation to increase demand for contraception and address barriers to contraceptive uptake
**Ankomah 2013**	Family planning interventions should concentrate on the benefits of family planning at the family level and not at the state or national level
**Aransiola 2014**	To significantly improve family planning adoption rates among urban slum dwellers in Nigeria, there is the need to specifically and specially target men alongside their female partners as well as other stakeholders who have significant influences at family and community level

*IUD* Intrauterine device, *BCC* Behavioural change communication, *EC* Education Campaign

## Discussion

The review has shown the uptake of family planning services and the challenges and barriers associated with poor uptake of services. Scaling up of family planning services has been a major challenge to reproductive health service providers in Nigeria. The country’s contraceptive prevalence rate (CPR) is still low at 17% [1]. Both health service and consumer factors have contributed in keeping the CPR low resulting in a huge unmet need for family planning and consequently, high unintended pregnancy rate [42]. The Uptake of family planning recorded in the reviewed studies ranges from 10.3 to 66.8%. Only three of the studies reviewed recorded an uptake of 50% or more. This is still poor and calls for targeted strategies to improve uptake.

Several challenges and barriers to uptake were reported by the studies included in this review. Both client and health service related factors were identified. Health service related factors identified include cost, prior counselling and difficulty accessing services. Others include procurement difficulties, long distances of sources, and poor service of Family Planning Clinics. Prior counseling was shown to significantly improve the continuation rate of contraception. A similar systematic review to determine the barriers of family planning and contraception services in sub-Saharan Africa identified similar challenges [7].

The issue of cost of family planning methods is an important challenge that must be addressed to improve the uptake of family planning services. Also, access to family planning services is another factor affecting uptake of services that was identified in this review. Ensuring access to and availability and affordability of good-quality methods of contraception is a major determinant for achieving universal access to sexual and reproductive health [43]. The findings of this review about the factors influencing uptake of family planning services offer some guidance for health planners about strategies that should be prioritized. The health service factors such as cost, and poor access to health services highlight the need for planners to implement strategies that reduce these access barriers. It is recommended that there is need to raise awareness about maternal health services, bring it closer to the mothers and make it more affordable.

The client factors identified such as misconceptions, fear of side effects, low education, uncertainty about its need, and ignorance of family planning and its sources shows the need for provision of information about family planning methods. Strategies to ensure that clients are supplied with necessary information about the different methods, and their potential side effects are important to improve uptake of family planning services [13, 29, 31]. Religious belief was identified by some of the studies as one of the challenges to uptake of family planning services. Program planners should develop strategies that target religious groups through engaging the religious leaders, this will go a long way in overcoming this barrier [15]. Partner disapproval was also pointed out by some of the studies. Strategies that target the men should be adopted by program implementers since men are the major decision makers in the African culture and specifically in Nigeria. It is also important to encourage spousal communication and joint decision making on health matters [16, 30]. This will contribute in addressing the challenges to use of family planning services. In addition, male friendly services should be included as part of family planning services [17].

A number of studies highlighted culture as a challenge to uptake of family planning services. This explains why most studies reported desire for more children as a challenge to family planning use. African women are considered responsible for increasing the family size and failure to do so attracts negative judgment from the society. In Africa, family planning is viewed as a means of restricting growth and economic productivity. Interventions in sub-Saharan Africa and in Nigeria specifically must consider the complex cultural and social norms of each group of individuals so as to identify strategies that will fit into each situation. Community attitude towards modern family planning is an important factor that may influence adoption of family planning by married women. Social and community support of family planning may help to promote adoption of family planning. Therefore, traditional rulers can be targeted to serve as advocates to address these deep seated beliefs.

Study limitation includes the use of few databases for search. However, this review draws its strength from the fact that it focused on a particular context and will therefore be of benefit for policy making in the specific regions.

## Conclusion

Challenges that were identified include lower education, desire for more children, side effect, uncertainty about its need, partner disapproval, previous side effects, religious beliefs, cost of services, and difficulty accessing services. A multi-sectoral approach including the traditional leaders, religious leaders, education sector, reproductive health professionals, and women affairs department among others is needed to address the multi pronged challenges facing uptake of family planning services.

## Supplementary Information

**Additional file 1.**

**Additional file 2.**

**Additional file 3.**

**Additional file 4.**

## Data Availability

All data relevant to the study are included in the article or uploaded as supplementary information.

## Abbreviations

EC: Emergency contraception
ART: Antiretroviral
HCT: HIV counseling & Testing
CPR: Contraceptive Prevalence Rate
