# Supplementary material for: Why does uptake of family planning services remain sub-optimal among Nigerian women? A systematic review of challenges and implications for policy
Source: Contracept Reprod Med. 2020 Oct 31;5:30. doi: 10.1186/s40834-020-00133-6 (PMC7603738; doi:10.1186/s40834-020-00133-6)
Supplement: Supplementary file 4 — Additional file 4. [file 40834_2020_133_MOESM4_ESM.docx]

CASP quality assessment result

|  | Yes | Can’t tell | No |
| --- | --- | --- | --- |
| Was there a clear  statement of the aims of  the research? | 4 |  |  |
| Is a qualitative  methodology  appropriate? | 4 |  |  |
| Was the research  design appropriate to  address the aims of the  research? | 4 |  |  |
| Was the recruitment  strategy  appropriate to  the aims of the  research? | 4 |  |  |
| Was the data collected in  a way that addressed the  research issue? | 4 |  |  |
| Has the relationship  between researcher and  participants been  adequately considered? | 3 | 1 |  |
| Have ethical issues been  taken into consideration? | 3 | 1 |  |
| Was the data analysis  sufficiently rigorous? | 4 |  |  |
| Is there a clear statement  of findings? | 4 |  |  |
| How valuable is the  research? | 4 |  |  |
